# Prognostic Significance of the Serum Creatinine Level During the Shock Stage in Severe Burn Patients: A 10-Year Retrospective Study

**DOI:** 10.1155/mi/8876691

**Published:** 2025-06-23

**Authors:** Wei Zhu, Xiaorong Xie, Ziqin Shu, Li Li, Gaozhong Hu, Huapei Song

**Affiliations:** Institute of Burn Research, The First Affiliated Hospital of Army Medical University, State Key Laboratory of Trauma and Chemical Poisoning, Chongqing 400038, China

**Keywords:** prognosis, serum creatinine (Scr), severe burns, shock stage

## Abstract

**Objective:** To evaluate the significance of serum creatinine (Scr) level during the shock stage as a prognostic indicator for 90-day mortality in severe burn patients.

**Methods:** This retrospective cohort study included 224 severe burn patients with a burn ≥50% total body surface area (TBSA) admitted to the First Affiliated Hospital of Army Medical University from January 1, 2014, to December 31, 2023. Patient demography, the burn severity, infection markers, protein levels, renal function indicators, and prognostic indicators, including the hospital 90-day mortality rate, were collected. The comparisons of these indicators between the survival and death groups were performed by means of the independent-samples Mann–Whitney *U* test and chi-square test. Then the significantly different indicators between the two groups were subjected to univariate and multivariate logistic regression analyses. The independent risk factors affecting the prognosis of severe burn patients during the shock stage were screened, and a nomogram for the prediction of the survival rate of severe burn patients was constructed using the indicators in the shock stage. A receiver operating characteristic (ROC) curve was drawn to obtain the corresponding cut-off value. Differences between the two groups of patients separated according to the cut-off value were analyzed. The difference between the survival rate of both groups of patients during hospitalization was analyzed using the Kaplan–Meier(K-M) curve.

**Results:** The survival rate was 183/224 with total length of stay (LOS) in hospital of 66 days (27–113). The differences in TBSA, burn index (BI), infection indicators (leukocytes, C-reactive protein [CRP], and procalcitonin [PCT]), and renal function indicators (Scr, blood urea nitrogen [BUN], and cystatin C [CysC]) between the survival group and the death group were significant (*p*  < 0.05). Logistic regression analysis revealed that the Scr level during the shock stage was an independent risk factor for the prognosis of the risk of death in severe burn patients with a cut-off value of 100 μmol/L. Compared with the low-Scr group (Scr < 100 μmol/L), the high-Scr group (Scr ≥ 100 μmol/L) had a larger TBSA and higher BI. The Scr level was positively correlated with the increase in the TBSA and BI. The development of persistent organ dysfunction (POD) and mortality in the high-Scr group was significantly greater than those in the low-Scr group.

**Conclusion:** The Scr level during the shock stage is an independent risk factor for hospital death, which is important for the prognosis of the 90-days' mortality of severe burn patients, in combination with patient age, TBSA, and BI.

## 1. Background

Severe burn patients with large and deep burns often have complications, such as shock, sepsis, and multiple organ dysfunction, with a high mortality rate [1]. Especially in the shock stage (within 48 h after burn injury), patients are prone to haemodynamic instability and acute organ failure, which is a critical period for the early treatment of burns [1, 2]. Acute kidney injury (AKI) is a common complication of severe burns, and the serum creatinine (Scr) level is a key indicator of renal function. Studies have shown that changes in Scr levels in burn patients are correlated with the severity of kidney injury and affect the prognosis of patients [3]. The Scr level increases with larger total body surface area (TBSA), suggesting that renal dysfunction is closely related to the severity of burns [4]. Burn patients (median 13% TBSA [8–24]) with elevated Scr levels at admission had a higher incidence of complications and mortality and a poorer prognosis [5]. However, there are few reports on the prognostic value of the Scr level during the shock stage in severe burn patients (≥50% TBSA). The purpose of this study was to retrospectively analyze the clinical data of severe burn patients enrolled in the largest burn center in Southwest China, and evaluate the potential value of the Scr level during the shock stage as a prognostic indicator.

## 2. Subjects and Methods

This retrospective cohort study was in line with the basic principles of the Declaration of Helsinki. This study was approved by the Ethics Committee of the First Affiliated Hospital of the Army Medical University of the Chinese People's Liberation Army (No. KY2024140). In this study, the patients' personal information was not disclosed, and written informed consent was not needed.

### 2.1. Inclusion Criteria

The inclusion criteria were as follows: age ≥18 years, admitted to the hospital within 24 h after injury, and burn area ≥ 50% TBSA. The exclusion criteria were as follows: special types of burns (electrical burns, hot crush injury, radiation burns, detonation injuries, chemical burns, etc.), patients with preexisting chronic kidney disease or renal insufficiency prior to injury, patients with severe systemic diseases (such as hypertension, diabetes, immune deficiency, and malignant tumors) before injury, patients who were voluntarily discharged from the hospital within 1 week after injury or who did not cooperate with treatments, and patients with incomplete information or missing key data.

### 2.2. Clinical Data

The following clinical baseline data from January 1, 2014, to December 31, 2023, were collected from the database of the First Affiliated Hospital of Army Medical University: demographic information (sex, age), time of injury, time of admission, causes of burn, severity of burn (BA, burn index [BI]), infection markers (leukocytes, neutrophil-to-lymphocyte ratio [NLR], C-reactive protein [CRP], procalcitonin [PCT]), protein levels (albumin, globulin), renal function indicators (Scr, blood urea nitrogen [BUN], cystatin C [CysC]), and prognostic indicators (length of stay [LOS] in hospital, LOS in the burn intensive care unit [BICU], mean daily cost in hospital, incidence of sepsis within 28 days, persistent organ dysfunction (POD), survival status within 90 days of injury or hospital admission). To record the transient fluctuations of the biomarkers, all the infection markers, protein levels, and renal function indicators were measured within 48 h postburn during the shock stage. To ensure accuracy and minimize variability, the two consecutive measurements taken within 48 h after admission were collected for analysis.

### 2.3. Grouping Criteria

Patients were divided into a survival group and a death group according to whether the patient died within 90 days of hospital admission.

A receiver operating characteristic (ROC) curve analysis was used to determine the predictive value of Scr for the risk of death in severe burn patients, and a cut-off value was determined. According to the cut-off value, patients were divided into a low-Scr group and a high-Scr group.

### 2.4. Sample Size Estimation

The sample size estimation for this study was performed using PASS 21 (Power Analysis and Sample Size Software, Version 21.0.3, NCSS LLC). Based on previous literature [5, 6], the matching ratio between high-Scr and low-Scr groups was approximately 1:3.5. The mortality rates were approximately 3.9% in the low-Scr group and 20%–40% (calculated as 30%) in the high-Scr group. Using PASS 21 software with *α* = 0.05 and *β* = 0.10, the calculated sample sizes were N1 = 24 for the high-Scr group and N2 = 84 for the low-Scr group, requiring a total sample size of 108 cases (N1 + N2). Considering a 20% loss-to-follow-up rate, the final required sample size was 30 + 105 = 135 cases.

### 2.5. Sepsis and POD Definitions

Sepsis: The sepsis criteria of the American Burn Association (ABA) were used [7].

POD: POD is defined as the presence of one or more of persistent circulatory failure as defined by the ongoing need for vasopressor agents, such as norepinephrine, epinephrine, vasopressin, ≥ 5 μg/kg/min of dopamine, or ≥ 50 μg/min phenylephrine for more than 2 h in a given day; persistent renal failure as defined by the need for any ongoing renal replacement therapy; or persistent respiratory/neuromuscular failure as defined by the ongoing need for mechanical ventilation (not including continuous positive airway pressure or noninvasive ventilation) at the outcome assessments time points [8].

### 2.6. Treatments

All patients were admitted to BICU. Fluid resuscitation treatment for burns was performed according to the fluid replacement formula proposed by the Army Medical University of the Chinese People's Liberation Army. The pulse-indicated continuous cardiac output (PiCCO) system was used for haemodynamic assessment. Patients with shock were given vasoactive drugs to maintain blood pressure; patients with severe inhalation injury or acute respiratory distress syndrome (ARDS) were given supportive treatments, such as tracheotomy and ventilator-assisted breathing, as well as treatments to maintain organ function and internal environment stability; and patients with acute kidney failure were given continuous renal replacement therapy (CRRT). Previously, we empirically used broad-spectrum antibiotics (e.g., imipenem/cilastatin sodium or cefoperazone/sulbactam sodium) for infection prophylaxis. However, since 2024, the use of prophylactic antibiotics during the shock phase is no longer recommended.

### 2.7. Data Analysis

SPSS 25.0 software was used for analysis, and R language was used for analysis and visualization. The numerical data are expressed as frequencies (percentages), and the chi-square test was performed. Non-normally distributed continuous data are expressed as medians and interquartile range (IQR), and the Mann–Whitney *U* test was used. Univariate and multivariate logistic regression analyses were performed on the indicators with statistical significance in the comparison, independent risk factors for the prognosis of severe burn patients during the shock stage were screened, and the nomogram of indicators in the shock stage for predicting survival in severe burn patients was plotted. ROC curves of independent risk factors for the prognosis prediction of severe burn patients were drawn, the cut-off value was obtained, the patients were divided into two groups according to the cut-off value, and the survival rates of the two groups were compared via Kaplan–Meier (K-M) survival curves. Spearman's correlation coefficients were obtained for the variables. The significance level of all the statistics was set to 0.05 (the significance level of the univariate logistic regression analysis was set to 0.1).

## 3. Results

A total of 224 severe burn patients were enrolled in this study, including 182 males and 42 females, with the age of 45 years (34–52), 69% TBSA (55–84), and BI was 53 (42–70). The hospital survival rate was 183/224 during 66 days (27–113) of stay in the hospital. (Tables 1 and 2).

### 3.1. Comparison of the Baseline Data and Indicators in Shock Stage Between the Survival Group and Death Group

**Figure 1:**
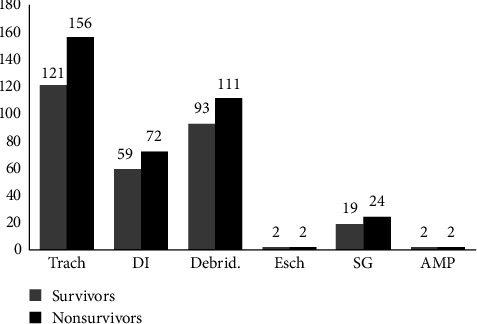
Predominant surgical interventions during shock stage. AMP, amputation; Debird, debridement; DI, decompressive incision; Esch, escharotomy/tangential excision; SG, skin grafting; Trach, tracheotomy.

At baseline, there were no statistically significant differences in gender, age, causes of burn, or time to hospital after injury between the survival group and the death group (*p*  > 0.05). However, the death group exhibited significantly larger TBSA (90% [81–95] vs. 63% [55–80]) and higher BI (77 [69–92] vs. 48 [40–63]) compared to the survival group (p  < 0.05). In terms of infection markers, the death group showed elevated WBC (22.76 × 10^9^/L [20.53–30.79] vs. 19.61 × 10^9^/L [14.31–25.24]) and PCT (4.94 ng/ml [1.84–10.35] vs. 1.85 ng/ml [0.65–4.13]) levels but paradoxically lower CRP (32.88 mg/L [10.17–58.74] vs. 47.78 mg/L [24.71–79.58]) levels compared to the survival group (all *p*  < 0.05), while no significant difference was observed in NLR between groups (*p*  > 0.05). Plasma protein profiles (albumin and globulin) revealed no significant intergroup differences (*p*  > 0.05). Regarding renal function, the death group demonstrated higher Scr (122 μmol/L [76–177] vs. 72 μmol/L [60–91]), BUN (7.96 mmol/L [6.83–10.61] vs. 6.26 mmol/L [4.98–8.20]), and CysC (0.95 mg/L [0.81–1.22] vs. 0.74 mg/L [0.57–0.92]) levels than the survival group (*p*  < 0.05). During the shock stage, the death group required significantly more frequent use of vasopressors (29% vs. 4%), hemodialysis (34% vs. 9%), and mechanical ventilation (80% vs. 31%), (all *p*  < 0.05), but no significant difference was found in surgical interventions (*p*  > 0.05) (Table 1). The predominant surgical interventions during the shock stage of severe burns were tracheotomy (Trach), debridement (Debird), decompressive incision (DI), skin grafting (SG), escharotomy/tangential excision (Esch), and amputation (AMP) (Figure 1).

### 3.2. Factors Affecting the Mortality of Severe Burn Patients During the Shock Stage

Univariate logistic regression analysis revealed that TBSA, BI, infection markers (leukocytes, CRP, and PCT), and renal function indicators (Scr and BUN) were risk factors for death in severe burn patients during the shock stage (*p*  < 0.01). Multivariate logistic regression analysis revealed that the Scr level during shock stage was an independent risk factor for death in severe burn patients (*p*  < 0.05) (Table 3). Of the 224 enrolled patients, 37 patients (16.5%) did not undergo CRP testing, and 16 patients (7.1%) did not undergo PCT testing during the shock stage. Patients were subjected to PCT and CRP tests only when they showed signs of infection. During the shock period, most patients had no visible signs of systemic infection, so they did not receive PCT and CRP tests. These patients were excluded from the univariate logistic regression analysis involving CRP and PCT.

### 3.3. Prediction of Mortality in Severe Burn Patients Using Shock Stage Indicators

**Figure 2:**
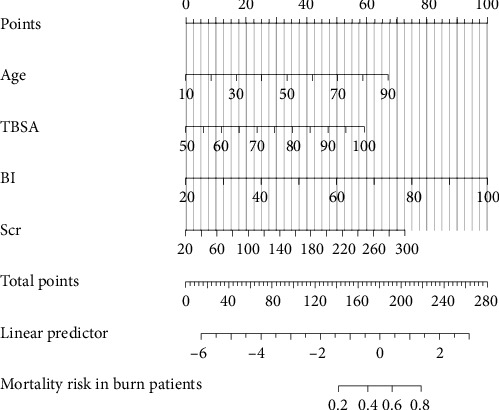
Nomogram for mortality risk in burn patients. BI, burn index; Scr, serum creatinine; TBSA, total body surface area.

**Figure 3:**
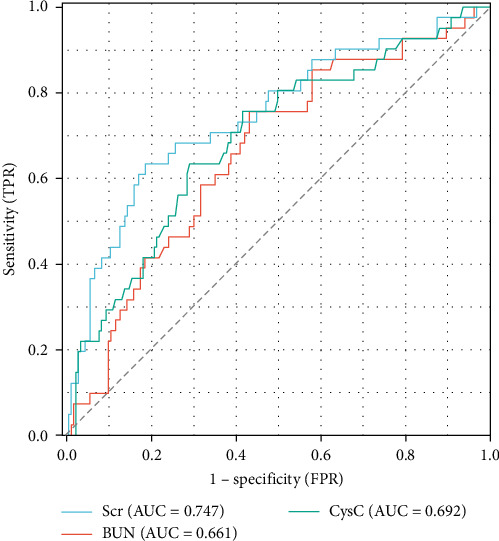
The ROC curve for the predictive value of renal function indicators for the risk of death in patients with severe burn. BUN, blood urea nitrogen; CysC, cystatin C; Scr, serum creatinine.

Previous studies have shown that age, TBSA, and BI are independent risk factors for death in burn patients [9]. Therefore, in this study, the Scr level during the shock stage was combined with these indicators, and a nomogram for the prediction of the survival rate of severe burn patients was constructed using these indicators during the shock stage (Figure 2). The results revealed that the older the severe burn patient was, the greater the TBSA and BI, the higher the Scr during the shock stage, and the higher the risk of death for the patient. The area under the ROC curve (AUC) of the predictive value of the Scr level during the shock stage for the risk of death of severe burn patients was 0.747 (95% confidence interval [CI]: 0.657–0.837), the cut-off was 100 μmol/L, the sensitivity was 63.42%, and the specificity was 81.42% (Figure 3 and Table 4).

### 3.4. Demographic and Epidemiological Data of Burn Patients in the High-Scr Group and the Low-Scr Group

**Figure 4:**
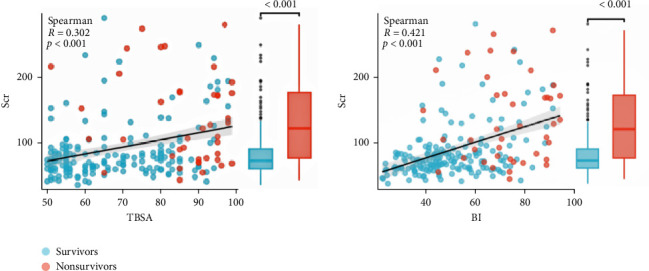
Bivariate correlations of Scr with TBSA and BI. BI, burn index; Scr, serum creatinine; TBSA, total body surface area.

According to the cut-off value of Scr, severe burn patients were divided into a low-Scr group (164 patients) and a high-Scr group (60 patients). There was no significant difference in age (43 years [33–51] vs. 47 years [36–54], *p*=0.10) or cause of burn (*χ*^2^ value = 0.885, *p*=0.347) between the two groups of patients. The severe burn patients were mainly male patients. Compared with low-Scr group, the high-Scr group had greater proportion of male patients (90%>78%, *p*  < 0.05), a larger TBSA (84% [65–94] > 62% [55–80], *p*  < 0.05) and a higher BI (71[56–85] > 47[40–63],*p*  < 0.05). The Scr level was positively correlated with the TBSA (Spearman *R* = 0.302, *p*  < 0.05) and BI (Spearman *R* = 0.421, *p*  < 0.05) (Figure 4). The time to hospital after injury in the high-Scr group (7.11 h [4.70–10.63] > 5.40 h [3.65–9.99], *p*  < 0.05) was longer than that in the low-Scr group (Table 2).

### 3.5. Comparison of Renal Function Indicators Between the High-Scr Group and the Low-Scr Group

**Figure 5:**
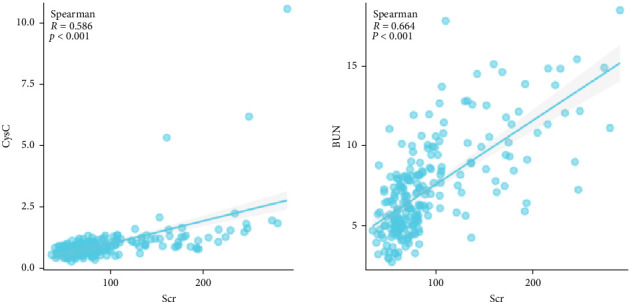
Bivariate correlations of Scr with BUN and CysC. BUN, blood urea nitrogen; CysC, cystatin C; Scr, serum creatinine.

The median Scr level of the high-Scr group was 154 μmol/L (122–194), and the low-Scr group was 68 μmol/L (58–80). The BUN level was greater in the high-Scr group (10.26 mmol/L [8.19–12.66] > 5.81 mmol/L [4.77–7.38], *p*  < 0.05), and the high-Scr group had higher CysC (1.14 mg/L [0.90–1.40] > 0.67 mg/L [0.54–0.86], *p*  < 0.05) (Table 2). Further analysis, as shown in Figure 5, revealed that CysC (Spearman *R* = 0.586, *p*  < 0.05) and BUN (Spearman *R* = 0.664, *p*  < 0.05) were strongly correlated with the trend of Scr. As common indicators reflecting renal function, these three indicators have certain reference value in predicting the prognosis of severe burn patients (Figure 3).

### 3.6. Prognosis in the High-Scr Group and the Low-Scr Group

**Figure 6:**
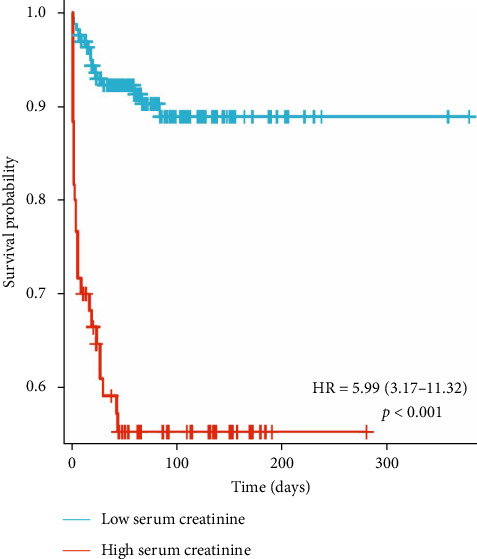
Kaplan–Meier analysis of survival outcomes by Scr levels in burn patients. Scr, serum creatinine.

There was no significant difference in the positive blood culture rate (*χ*^2^ value = 0.009, *p*=0.922) or the incidence of sepsis (*χ*^2^ value = 0.006, *p*=0.939) between the two groups. The median LOS in the high-Scr group 44 days (6–115) was significantly shorter than that in the low-Scr group 72 days (38–113), and the difference was significant (*p*  < 0.05); the daily hospitalization cost was higher in the high-Scr group (16.8 1000 RMB [renminbi] yuan [10.5–28.1] > 8.3 1000 RMB yuan [6.1–12.8], *p*  < 0.05); the incidence of POD in the high-Scr group was much higher than that in the low-Scr group (85% > 45%, *p*  < 0.05); and the high-Scr group had a higher risk of death (43.33% > 9.15%, *p*  < 0.05) (hazard ratio [HR] = 5.99, 95% CI: 3.17–11.32, *p*  < 0.05) (Table 2 and Figure 6).

## 4. Discussion

Scr is an end product of human muscle metabolism, which is excreted mainly by glomerular filtration, and is often used as an indicator of renal function. Under normal circumstances, the Scr level remains stable, but when renal function is impaired, the Scr excretion is blocked, resulting in elevated Scr levels. During the shock stage of severe burn patients, due to insufficient blood volume, tissue damage, and systemic inflammation, renal function may be damaged to varying degrees, which may cause changes in Scr levels. Kim et al. [10] reported that the Scr level was the best biomarker for diagnosis within 1 week of AKI. Previous studies on the changes in Scr in the early stage of burns have focused mainly on patients with small burns; however, the relationship between the Scr level and prognosis in severe burn patients during the shock stage is still unclear. This study revealed that the Scr level during the shock stage was an independent risk factor for death in severe burn patients. The ROC curve of death risk prediction for severe burn patients using the Scr level during the shock stage obtained a cut-off value of Scr at 100μmol/L, suggesting that patients with Scr levels higher than this cut-off value had a poorer prognosis and higher mortality. Moreover, the median Scr level in the low-Scr group was 68 μmol/L (close to the lower limit of the Scr test), whereas the median Scr level in the high-Scr group was 154 μmol/L (far higher than the upper limit of the Scr test), and this finding is in agreement with the clinical phenomenon that the Scr level is lower during the shock stage in severe burn patients with a good prognosis.

Severe burns trigger a hypermetabolic state mediated by neuroendocrine activation and proinflammatory cascades, which accelerates proteolysis in skeletal muscles, particularly in full-thickness burns, leading to enhanced Scr synthesis [1, 11, 12]. Concurrently, hypovolemia induced renal hypoperfusion combined with myoglobinuric tubular injury significantly compromises glomerular filtration capacity [13, 14]. This pathophysiological duality of upregulated Scr generation coupled with impaired renal excretory function creates a metabolic disequilibrium, ultimately causing Scr accumulation that exceeds physiological clearance thresholds. Most non-AKI patients with severe burns often exhibit low Scr levels during the shock stage. It may be attributed to early aggressive fluid resuscitation, which mitigates renal hypoperfusion and preserves glomerular filtration. Furthermore, extravasation of creatinine from burn wounds may decrease the serum levels of Scr. Therefore, if the Scr level is abnormally increased in the early stage of burn injury, delayed resuscitation or massive destruction of muscle cells by deep burns, which can lead to AKI and poor prognosis, should be considered. This study also revealed that the TBSA and BI of patients in the group were significantly lower than those in the high-Scr group, and the increase in Scr was positively correlated with the increases in TBSA and BI, indicating that the risk of death can be predicted from Scr level combined with TBSA and BI during the shock stage in severe burn patients. In addition, this study revealed that patients in the high-Scr group were hospitalized longer after injury, which may be related to AKI caused by insufficient early fluid resuscitation or deep burns. This study also revealed that there was a significant difference between the two groups in terms of sex. The proportion of males in the high-Scr group was greater than that in the low-Scr group, which was consistent with the findings of previous studies, indicating that sex hormones are related to Scr levels, and testosterone can promote the increase in Scr levels, whereas estradiol can reduce Scr levels [15].

Some recent studies have used the combination of POD and mortality as a composite endpoint in clinical trials of critically ill patients [8]. This study used POD + Death as a prognostic indicator and revealed that the incidences of POD and mortality in the high-Scr group were significantly higher than those in the low-Scr group, which further confirmed the value of the Scr level in predicting the prognosis of severe burn patients. These findings are consistent with previous studies [4, 5]. Moreover, the daily hospitalization cost in the high-Scr group was higher than that in the low-Scr group, but the LOS was shorter. The possible reason is that the high-Scr group was more critically ill and had a higher mortality rate, resulting in a lower median LOS for patients. However, there was no significant difference between the two groups in terms of the positive blood culture rate or the 28-day incidence of sepsis, which is slightly different from the previous study [5]. This may be related to the fact that severe burn patients are at high risk of developing sepsis.

Studies have shown that CysC can be used as a biomarker of AKI in burn patients and can predict kidney injury and recovery in patients earlier than Scr [16]. Seki et al. [17] revealed that higher BUN levels were associated with adverse renal outcomes independent of the estimated GFR, indicating that BUN may be a useful marker for predicting the progression of kidney disease. The results of this study revealed strong correlations between Scr and BUN, and CysC during the shock stage, and both BUN and CysC had certain predictive value for evaluating the prognosis of burn patients. The Scr level had a higher predictive value for the risk of death in severe burn patients during the shock stage. Scr outperformed BUN and CysC in mortality prediction is likely due to its direct reflection of glomerular GFR [17]. While BUN is influenced by nonrenal factors (e.g., protein intake, catabolism, and inflammation etc.), and CysC, though less variable, may be affected by thyroid dysfunction, Scr remains a more stable marker of renal filtration in the acute phase [18, 19]. Moreover, Scr elevation during the shock stage reflects not only renal hypoperfusion but also burn depth and magnitude of muscle destruction [13, 14]. Massive burns induce higher catabolism with profound proteolysis, releasing creatinine precursors proportional to the extent of necrotic tissue (Spearman *R* = 0.421 with BI, *p*  < 0.05). Thus, higher Scr levels (≥100 μmol/L) signify dual pathology: inadequate resuscitation (reduced GFR) and extensive tissue injury. And higher Scr levels are associated with increased mortality (HR = 5.99; 95% CI, CI: 3.17–11.32). In contrast, BUN and CysC may rise later or resolve with resuscitation, reducing their prognostic specificity during the shock stage.

This retrospective cohort study has several limitations. First, this was a single-center study, and the results may be affected by the specific medical environment and patient population, limiting the general applicability of the study results. Second, the interference of some potential influencing factors cannot be completely excluded through the analysis of the previous data. Third, it is noteworthy that the diagnostic criteria for burn sepsis in this study are based on the consensus first published by the ABA in 2007 [7]. Clinically, burn sepsis has specific characteristics, the definition of which is different from sepsis 3.0 [20]. Nevertheless, burns have always been excluded from the Surviving Sepsis Campaign [21], which has been developed to improve outcomes for all patients with sepsis. Therefore, most studies about burn sepsis are mainly based on the diagnostic criteria of ABA. In 2023, the International Society for Burn Injuries (ISBI) developed the Surviving Sepsis After Burn Campaign with the goal of improving the outcome of sepsis in burn patients [22]. The diagnostic criteria of burn sepsis proposed by ISBI might be used in future studies. In addition, the sample size of this study was relatively small, and there may be some bias. The conclusions of this study need to be validated with larger multicentre studies in the future.

## 5. Conclusion

In summary, this study confirms that the Scr level during the shock stage is an independent risk factor for the prognosis of death in severe burns. When the patient's Scr level exceeds the cut-off value (100 μmol/L), the patient's prognosis is poor, and timely intervention treatment should be applied. The Scr level combined with age, TBSA, and BI is important for evaluating the prognosis of severe burn patients. Future studies need to explore the combined application of Scr and other biomarkers in a more extensive patient population.

## Figures and Tables

**Table 1:** Differences in shock stage parameters between the burn survival and death groups.

Parameters	Survival status		Total (*n* = 224)	Test value	*p*-Value
	Survivors (*n* = 183)	Nonsurvivors (*n* = 41)			
Sex (*n* [%])					
Female	33 (18.03)	9 (21.95)	42 (18.75)	0.338	0.561^a^
Male	150 (81.97)	32(78.05)	182 (84.21)		
Age (years, [median, IQR])	45 (34–52)	45 (35–55)	45 (34–52)	−0.828	0.408^b^
Causes of burn (*n* [%])					
Flame	165 (90.16)	35 (85.37)	200 (89.29)	0.806	0.369^a^
Scald	18 (9.84)	6 (14.63)	24 (10.71)		
TBSA (%, [median, IQR])	63 (55–80)	90 (81–95)	69 (55–84)	−6.822	<0.001^b^
BI (median, IQR)	48 (40–63)	77 (69–92)	53 (42–70)	−7.054	<0.001^b^
Time to hospital after injury (hours, [median, IQR])	5.97 (4.00–10.69)	6.00 (4.15–8.72)	5.98 (4.00–10.16)	0.247	0.805^b^
Infection markers					
WBC (10^9/L, [median, IQR])	19.61 (14.31–25.24)	22.76 (20.53–30.79)	20.75 (15.77–26.71)	−3.094	0.002^b^
NLR (median, IQR)	13.73 (10.42–18.81)	15.84 (10.79–20.25)	14.58 (10.92–19.87)	−0.785	0.432^b^
CRP (mg/L, [median, IQR])	47.78 (24.71–79.58)	32.88 (10.17–58.74)	45.78 (24.76–78.92)	2.186	0.029^b^
PCT (ng/ml, [median, IQR])	1.85 (0.65–4.13)	4.94 (1.84–10.35)	2.08 (0.71–5.41)	−3.439	<0.001^b^
Plasma protein					
Alb (g/L, [median, IQR])	27.57 (24.68–30.73)	26.50 (23.60–28.90)	27.43 (24.28–30.72)	1.192	0.233^b^
Glb (g/L, [median, IQR])	21.93 (19.07–24.40)	20.83 (17.95–23.83)	21.75 (18.90–24.30)	1.494	0.135^b^
Renal function					
Scr (μmol/L, [median, IQR])	72 (60–91)	122 (76–177)	76 (61–105)	−4.942	<0.001^b^
BUN (mmol/L, [median, IQR])	6.26 (4.98–8.20)	7.96 (6.83–10.61)	6.62 (5.07–8.65)	−3.218	<0.001^b^
CysC (mg/L, [median, IQR])	0.74 (0.57–0.92)	0.95 (0.81–1.22)	0.78 (0.58–0.99)	−3.837	<0.001^b^
Treatment					
Vasopressors (*n* [%])					
No	175 (95.63)	29 (70.73)	204 (91.07)	25.533	<0.001^a^
Yes	8 (4.37)	12 (29.27)	20 (8.93)		
Hemodialysis (*n* [%])					
No	166 (90.71)	27 (65.85)	193 (86.16)	17.356	<0.001^a^
Yes	17 (9.29)	14 (34.15)	31 (13.84)		
Mechanical ventilation (*n* [%])					
No	126 (68.85)	8 (19.51)	134 (59.82)	33.926	<0.001^a^
Yes	57 (31.15)	33 (80.49)	90 (40.18)		
Surgical (*n* [%])					
No	30 (16.39)	2 (4.88)	32 (14.29)	3.627	0.057^a^
Yes	153 (83.61)	39 (95.12)	192 (85.71)		

*Note:* WBC, white blood cell count.

Abbreviations: Alb, albumin; BI, burn index; BUN, blood urea nitrogen; CRP, C-reactive protein; CysC, cystatin C; Glb, globulin; NLR, neutrophil-to-lymphocyte ratio; PCT, procalcitonin; Scr, serum creatinine; TBSA, total body surface area.

^a^Chi-square test.

^b^Mann–Whitney U test.

**Table 2:** Clinical characteristics of burn patients stratified by serum creatinine levels.

Characteristics	Serum creatinine		Total (*n* = 224)	Test value	*p*-Value
	Low (*n* = 164)	High (*n* = 60)			
Sex (*n* [%])					
Female	36 (21.95)	6 (10.00)	42 (18.75)	4.119	0.042^a^
Male	128 (78.05)	54 (90.00)	182 (84.21)		
TBSA (%, [median, IQR])	62 (55–80)	84 (65–94)	69 (55–85)	5.480	<0.001^b^
BI (median, IQR)	47 (40–63)	71 (56–85)	53 (42–70)	6.505	<0.001^b^
Time to hospital after injury (hours, [median, IQR])	5.40 (3.65–9.99)	7.11 (4.70–10.63)	5.98 (4.00–10.20)	2.055	0.040^b^
Renal function					
Scr (μmol/L, [median, IQR])	68 (58–80)	154 (122–194)	76 (61–105)	11.454	<0.001^b^
BUN (mmol/L, [median, IQR])	5.81 (4.77–7.38)	10.26 (8.19–12.66)	6.62 (5.07–8.66)	8.855	<0.001^b^
CysC (mg/L, [median, IQR])	0.67 (0.54–0.86)	1.14 (0.90–1.40)	0.78 (0.58–0.99)	8.637	<0.001^b^
Outcomes					
LOS (days, [median, IQR])	72 (38–113)	44 (6–115)	66 (27–113)	−2.581	0.010^b^
Daily hospitalization cost (thousand RMB yuan, [median, IQR])	8.3 (6.1–12.8)	16.8 (10.5–28.1)	10.2 (6.6–17.5)	5.799	<0.001^b^
POD (*n* [%])					
Yes	74 (45.12)	51 (85.00)	125 (55.80)	28.325	<0.001^a^
No	90 (58.88)	9 (15.00)	99 (44.20)		
Survival status (*n* [%])					
Survivors	149 (90.85)	34 (56.67)	183 (81.70)	34.334	<0.001^a^
Nonsurvivors	15 (9.15)	26 (43.33)	41 (18.30)		

Abbreviations: BI, burn index; BUN, blood urea nitrogen; CysC, cystatin C; LOS, length of stay; POD, persistent organ dysfunction; RMB, renminbi; Scr, serum creatinine; TBSA, total body surface area.

^a^Chi-square test.

^b^Mann–Whitney U test.

**Table 3:** Risk factors for survival of severe burn patients analyzed by logistic regression.

Parameters	Total (*n*)	Univariate analysis		Multivariate analysis	
		Odds ratio (95% CI)	*p*-Value	Odds ratio (95% CI)	*p*-Value
TBSA	224	1.101 (1.067–1.137)	< 0.001	1.053 (0.968–1.147)	0.230
BI	224	1.086 (1.059–1.114)	< 0.001	1.045 (0.973–1.123)	0.226
WBC	224	1.051 (1.012–1.092)	0.010	0.986 (0.919–1.056)	0.682
CRP	187	0.989 (0.979–1.000)	0.042	0.989 (0.975–1.004)	0.157
PCT	208	1.024 (0.999–1.049)	0.057	0.965 (0.925–1.007)	0.104
Scr	224	1.016 (1.010–1.023)	< 0.001	1.019 (1.005–1.034)	0.008
BUN	224	1.161 (1.046–1.290)	0.005	0.862 (0.679–1.094)	0.222
CysC	224	1.184 (0.858–1.633)	0.304	—	—

*Note:* Some patients did not undergo CRP and PCT testing during the shock stage; these patients were not included in the single-factor analysis. WBC, white blood cell count.

Abbreviations: BI, burn index; BUN, blood urea nitrogen; CRP, C-reactive protein; Cys C, cystatin C; PCT, procalcitonin; Scr, serum creatinine; TBSA, total body surface area.

**Table 4:** Renal function indicators for predicting mortality risk in patients with severe burns.

Variables	AUC	CI	Cut-off value	Sensitivity	Specificity
Scr	0.747	0.657–0.837	100.21	0.6342	0.8142
BUN	0.661	0.570–0.752	6.825	0.7561	0.5683
CysC	0.692	0.600–0.784	0.885	0.7104	0.6964

Abbreviations: AUC, area under the curve; BUN, blood urea nitrogen; CI, confidence interval; CysC, cystatin C; Scr, serum creatinine.

## Data Availability

The data that support the findings of this study are available from the corresponding author upon reasonable request.
